# Perioperative antibiotic prescribing in two private sector hospitals in central India

**DOI:** 10.1186/2047-2994-4-S1-P176

**Published:** 2015-06-16

**Authors:** J Sparrentoft, M Sharma

**Affiliations:** 1 Department of Public Health Sciences, Karolinska Institutet, Sweden

## Introduction

Antibiotic resistance is increasing globally and high use of antibiotics has been found to correlate with increasing antibiotic resistance. Surgery related prophylactic use of antibiotics is common due to the high risk for patients to develop surgical site infections. In India, the world’s second most populated country there is currently no national prescribing guidelines to be implemented and data, both on overall and prophylactic use antibiotics in surgery is scarce.

## Objectives

To analyse the perioperative antibiotic prescribing among patients admitted with surgery indications at surgery departments of two tertiary care hospitals, a teaching hospital (TH) and a non-teaching (NTH).

## Methods

The study was conducted in Ujjain, Madhya Pradesh, India. Patients admitted between April 2008 and March 2011 at the surgery wards of the CR. Gardi Hospital (TH) and Ujjain Charitable Trust Hospital (NTH) were included in the study, 6171 from the TH and 6263 from the NTH. Four diagnosis groups were devised, upper/lower urinary tract and routine/emergency abdominal surgery indications. Patients with simultaneous infections were excluded. Demographic information and antibiotic prescribing data were analysed. DDDs were used to compare dosages. Adherence to the WHO list of essential medicines (WHOLEM) and National list of essential medicines of India (NLEMI) were analysed.

## Results

At the TH 88% and at the NTH 86% of patients were prescribed antibiotics. The most commonly prescribed antibiotics were imidazole derivates and quinolones at the TH and cephalosporins and antibiotic fixed dose combinations at the NTH. The average duration of hospital stay and duration of antibiotic treatment were 10.0 and 7.6 days at the TH and 3.9 and 3.2 days at the NTH. The overall average DDD/100 patient days were 72.5 in the TH and 110.5 at the NTH. Twenty five percent of prescriptions at the TH and 6% at the NTH were done using generic names. Adherence to WHOLEM and NLEMI were seen in 66% and 75% of prescriptions at the TH and 42% and 57% of prescriptions at the NTH.

## Conclusion

The result from the study indicates that a large proportion of patients admitted at surgery wards of the study hospitals were prescribed prophylactic antibiotics. It also indicates that the antibiotics are being prescribed in long courses and that there are large differences in the dosages prescribed.

## Disclosure of interest

None declared.

